# Capsular Contracture After Breast Augmentation: A Systematic Review and Meta-Analysis

**DOI:** 10.1093/asjof/ojaf003

**Published:** 2025-01-15

**Authors:** Evan Haas, Neophytos Christodoulou, Murilo Secanho, George Kokosis, Rafael D Malgor, Julian Winocour, Jason W Yu, David W Mathes, Christodoulos Kaoutzanis

## Abstract

Capsular contracture is characterized by the formation of a fibrous capsule around a breast implant after an augmentation mammaplasty, and often results in pain, firmness, and implant distortion. The aim of this meta-analysis was to investigate how implant and surgical characteristics affect rates of capsular contracture after breast augmentation. A systematic review and meta-analysis were performed in PubMed MEDLINE, EMBASE (OvidSP), and Cochrane Library. Comparison groups included smooth vs textured implants; subpectoral vs prepectoral implant placement; saline vs silicone implants. Odds ratios (ORs) were calculated for capsular contracture for each of these groups. The inclusion criteria were met in 24 studies. Smooth implants were associated with significantly higher capsular contracture rates compared with textured implants (OR = 2.80, 95% CI, 1.92-4.08). Subpectoral implant placement demonstrated significantly reduced capsular contracture rates compared with prepectoral placement (OR = 0.35, 95% CI, 0.25-0.50). No significant difference in capsular contracture rates was found between silicone and saline (OR = 0.39, 95% CI, 0.02-6.69). This meta-analysis suggests that textured-surface implants are associated with lower capsular contracture rates than smooth implants following breast augmentation. Additionally, subpectoral implant placement was associated with significantly reduced rates of capsular contracture compared with prepectoral placement. There was no significant difference in capsular contracture rates between saline and silicone implants. However, the absence of large, randomized controlled trials included in this study underscores the need for prospective investigation of the relationship between implant and surgical characteristics and postoperative outcomes.

**Level of Evidence: 2 (Risk):**

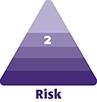

Augmentation mammaplasty, commonly known as breast augmentation surgery, consistently ranks as one of the most frequently performed aesthetic surgical procedures. In 2023 alone, just under 250,000 breast augmentations were performed by plastic surgeons in the United States.^1^ Although augmentations are elective procedures, they require the implantation of foreign devices into a patient's body and have been associated with several complications including infection, seroma and hematoma formation, implant malposition, sensory changes, and capsular contracture.^2^ Capsular contracture is one of the most common long-term complications and indications for reoperation after breast augmentation. It is thought to be caused by the body's inflammatory response, in which a thick, fibrotic capsule forms around the implant. It often leads to persistent pain, hardening, and distortion of the breast.^3^ Contracture is typically graded using the Baker Classification, with patients graded Class III or IV being those who usually require intervention.^4^

Because augmentations are quite common, there have been many advancements made in the surgical techniques and materials used in surgery to reduce the risk of complications and improve outcomes. Today, patients and surgeons can choose implants based on many criteria, including size, contour, texture of the implant's surface (textured vs smooth), plane of implant placement (prepectoral vs subpectoral), and the type of implant filler (silicone vs saline), many of which are thought to influence surgical outcomes and rates of capsular contracture.^5^

Regarding the surface texture, rough or textured implants are thought to force interruptions in the formation of the body's capsule, which may lead to lower rates of implant rippling and capsular contracture when compared with traditional smooth implants.^6,7^ A previous meta-analysis reported that the risk of capsular contracture when using smooth implants could be 4.67 times greater than the risk of contracture when using textured implants 1 year after breast augmentation.^8^ However, some publications have pointed out that the higher rates of infection seen in the use of textured implants, potentially because of increased growth of bacterial biofilm, may lead to a greater risk of contracture.^9^ Additionally, textured implants have been implicated in the development of breast implant-associated anaplastic large cell lymphoma.^10^

The plane of implant placement is also a subject of debate within the realm of breast augmentation techniques. Implants are frequently placed into the subpectoral dual plane pocket, which is partially covered by the pectoralis major muscle.^11,12^ Subpectoral implant placement has been shown to reduce rates of capsular contracture compared with prepectoral placement, potentially because it minimizes contact with the surrounding glandular tissue.^13,14^ Placement under the muscle may also produce superior aesthetic results because it reduces implant visibility, palpability, and rippling compared with prepectoral placement, which has limited coverage from the overlying soft tissues.^15^ Nevertheless, subpectoral placement of the implant can lead to breast animation deformity, resulting in patient dissatisfaction.^16^

Lastly, filler material is another implant characteristic to consider when discussing the risks of capsular contracture. Both silicone and saline implants have been used extensively in breast augmentation, but there is little consensus on which produces better surgical outcomes and rates of capsular contracture.^13,17^

The existing literature surrounding the impact of implant characteristics on rates of capsular contracture after breast augmentation, distinct from breast reconstruction, is rather limited. The relevant publications are often dated and lack a comprehensive analysis of all factors within this meta-analysis, which includes implant surface type, plane of implant placement, and the implant filler material.

## METHODS

### Search Approach

A systematic review of the literature and meta-analysis were conducted, as per the Preferred Reporting Items for Systematic Reviews and Meta-Analysis guidelines.^18^ The Prospero Registration Number of this study is CRD42024529482. Three databases were used for the literature search: Cochrane Library, EMBASE (OvidSP), and PubMed MEDLINE. The literature search covered the period from January 1947 to December 2023. The details of the literature search strategy are outlined in Supplemental Table 1. References within articles were also searched.

### Selection Criteria

Studies were included in the analysis if

- breast augmentation included the use of implants;
- primary procedures;
- clear definition of capsular contracture as Baker Grade III or IV;
- at least 1 of the 3 comparison groups was compared (smooth vs textured implants; prepectoral vs subpectoral placement of the implant; and silicone vs saline implants);
- published as a full-text article.

Studies were excluded if

- capsular contracture was not clearly defined or included Baker Grades I and II;
- breast reconstruction with implants;
- systematic reviews/meta-analyses;
- clinical trials not yet completed, letter to the editors, abstracts, no comparison groups, no report of capsular contracture, reviews, and not in the English language.

### Data Extraction

Three reviewers (E.H., M.S., and N.C.) determined the eligibility of all retrieved studies independently by screening the titles and abstracts, and then by full text for all potential studies identified. Any discrepancies were resolved through discussion with the seniors (C.K., D.W.M., G.K., J.W., J.W.Y., and R.D.M.). The data from each included study were extracted, listing the first author and year of publication, number of cases (breasts/patients), capsular contracture incidence, patient demographics (age, BMI, smoking), and follow-up. Risk of bias was assessed using the Cochrane Collaboration's risk of bias tool; high, low, or uncertain risk of bias. Risk domains included: selection bias, performance bias, detection bias, attrition bias, reporting bias, and vested interest bias.^19^

### Statistical Analysis

Because of the heterogeneous nature of the included studies, the Mantel–Haenszel statistical method was used for capsular contracture (dichotomous data). Odds ratios (ORs) were calculated at 95% CIs. Dispersion of observed and true effects among studies was assessed using Tau^2^ and *I*^2^ tests. Interpretation of *I*^2^ values was done as per Cochrane Handbook for Systematic Reviews of Interventions Version 6.3;^19^ 0% to 40% representing low heterogeneity; 30% to 60% representing moderate heterogeneity; 50% to 90% representing substantial heterogeneity; and 75% to 100% representing considerable heterogeneity. All statistical analyses were performed by ReviewManager 5.4.1.

## RESULTS

### Study Selection

**Figure 1.**
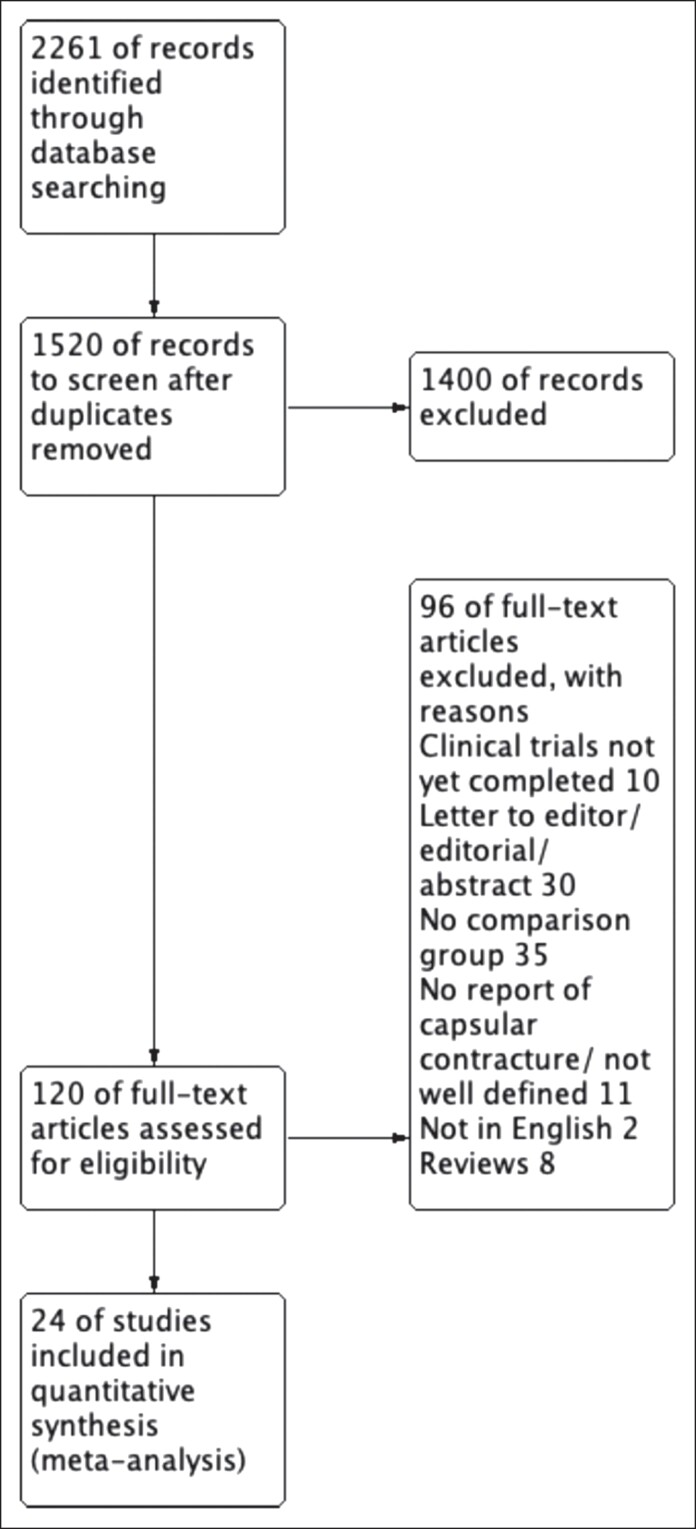
Preferred Reporting Items for Systematic Reviews and Meta-Analysis flow diagram.

A total of 2261 studies were identified using Cochrane Library, EMBASE (OvidSP), and PubMed MEDLINE. The inclusion criteria were met in 24 studies for the synthesis of this meta-analysis. The study flow diagram is illustrated in Figure 1. Table 1 summarizes the study characteristics and patient demographics.

**Table 1.** Study Characteristics and Patient Demographics of Included Studies

Study, year	Country	Design	No. of cases	Mean/median age, years		BMI, kg/m^2^		Smoking (%)		Mean/median follow-up, months		Implant manufacturer
				SM	TEX	SM	TEX	SM	TEX	SM	TEX	
Asplund, 1996	Sweden	P	110	30.0		NA		NA		12.0		Dow Corning
Burkhardt, 1994	USA	P	90	34.0		NA		NA		21.0		Mentor
Burkhardt, 1995	USA	P	104	33.0		NA		NA		20.0		McGhan
Calobrace, 2018*	USA	P	5122	NA		NA		NA		NA		NA
Coleman, 1991	UK	P	100	NA		NA		NA		NA		Mentor
Ersek, 1991	USA	P	400	NA		NA		NA		36.0	12.0	Surgitek, Mentor, Heyer-Schulte, Dow Corning
Filiciani, 2022	Argentina	R	506	33.5	33.4	NA		NA		24.0		Mentor, Nagor, Allergan, Eurosilicone
Hakelius, 1992	Sweden	P	50	31.0		NA		NA		12.0		McGhan
Henriksen, 2005*	Denmark	P	4499	33.0		NA		NA		19.5		Dow Corning
Lista, 2020	Canada	R	526^p^	36.3	36.0	23.9	24.0	23 (10.8)	52 (16.6)	15.4	25.2	Allergan, Mentor
Malata, 1997	UK	P	49^p^	NA		NA		NA		36.0		Mentor
Poeppl, 2007	Germany	P	48	40.0		NA		NA		NA		Mentor
Pollock, 1993	USA	R	197^p^	NA		NA		NA		21.5	16.5	Mentor
Spear, 2014*	USA	P	455^p^	34.0		20.7		NA		NA		Sientra
Stevens, 2013*	USA	P	5109	36.0		20.8		NA		60.0		Sientra
Tarpila, 1997	Sweden	P	42	33.0		NA		NA		12.0		McGhan
				SP	PP	SP	PP	SP	PP	SP	PP	
Benito-Ruiz, 2017	Spain	R	373^p^	NA		NA		NA		60.0		McGhan
Calobrace, 2018*	USA	P	5122	NA		NA		NA		NA		NA
Henriksen, 2005*	Denmark	P	4484	33.0		NA		NA		19.5		NA
Khan, 2013	UK	R	861^p^	33.0	30.9	NA		NA		36.0 (minimum)		Perthese, Poly Implant Prothese, McGhan, Allergan, Nagor
Pereira, 2009	Brazil	P	53^p^	26.1	24.5	NA		NA		NA		NA
Puckett, 1987	USA	P	192	30.0	33.0	NA		NA		20.0	34.0	Dow Corning
Shi, 2015	China	R	248	31.2	38.1	19.2	20.2	12 (11.4)	2 (5.1)	31.7	32.5	NA
Spear, 2014*	USA	P	452^p^	34.0		20.7		NA		NA		Allergan's Natrelle
Stevens, 2013*	USA	P	5109	36.0		20.8		NA		60.0		Sientra
Stutman, 2012*	USA	R	617^p^	NA		NA		101 (16.3)		28.8		Mentor
Vazquez, 1987	USA	R	196	31.9		NA		NA		17.4	38.1	Surgitek, Dow Corning, Heyer-Schulte
				SAL	SIL	SAL	SIL	SAL	SIL	SAL	SIL	
Cairns, 1980	South Africa	R	79	NA		NA		NA		NA		Heyer-Schulte
Henriksen, 2005*	Denmark	P	3502	33.0		NA		NA		19.5		NA
Stutman, 2012*	USA	R	617^p^	NA		NA		101 (16.3)		28.8		Mentor

Studies marked with an asterisk (*) report data for >1 comparison group. Studies marked with superscript “p” report case numbers as patient numbers. NA, not available; P, prospective; PP, prepectoral; R, retrospective; SAL, saline; SIL, silicone; SM, smooth; SP, subpectoral; TEX, textured.

**Figure 2.**
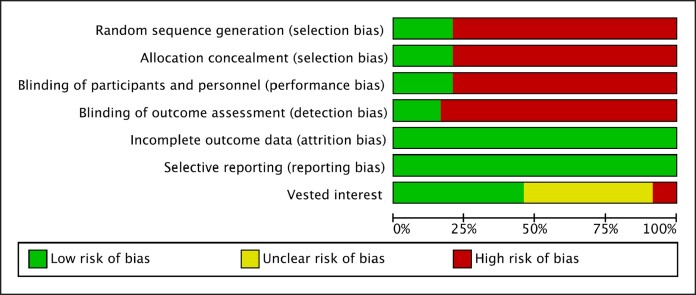
Risk of bias summary for included studies.

Figure 2 depicts the risk of bias (presented as percentages) and Supplemental Figure 1 describes the risk of bias for each individual study. Most of the included studies were high for selection, performance, and detection bias, except 4^20-23^ which were randomized and double-blinded trials. Attrition and reporting bias were low for all included studies. Two of the included studies were high for vested interest bias.^24,25^ The study by Spear et al was designed and funded by Allergan (breast implant manufacturer), and one of the authors of Stutman et al served as a consultant to Mentor (breast implant manufacturer) and also received education grants from them. The remaining studies had a low or uncertain risk of vested interest bias.

### Smooth vs Textured Implants

**Figure 3.**
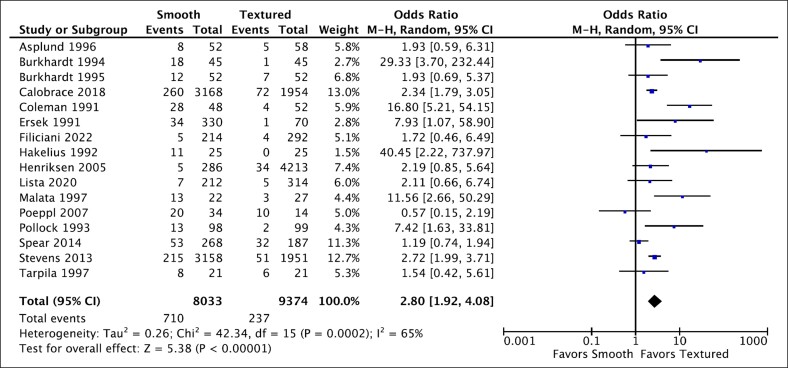
Forest plot for smooth vs textured.

Data on capsular contracture comparing smooth vs textured breast implants were obtained from 16 studies (17,407 cases).^20-24,26-36^ Smooth implants had significantly higher capsular contracture rates compared with textured implants (Figure 3, OR = 2.80, *P* < .00001 [95% CI, 1.92-4.08]). Substantial variation across these studies was observed (*I*^2^ = 65%).

### Subpectoral vs Prepectoral Placement of the Implant

**Figure 4.**
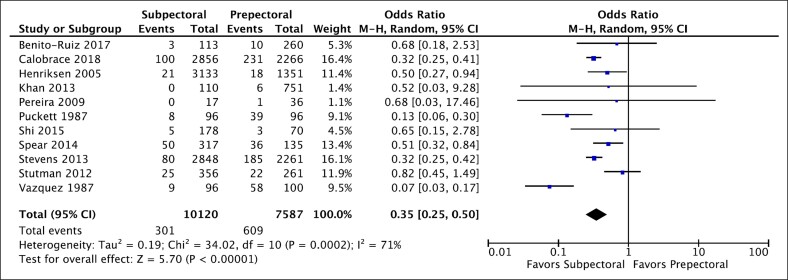
Forest plot for subpectoral vs prepectoral.

Data on capsular contracture comparing the subpectoral vs the prepectoral plane were obtained from 11 studies (17,707 cases).^24,25,28,35-42^ Subpectoral placement of the implant was associated with significantly lower capsular contracture rates compared with prepectoral placement (Figure 4, OR = 0.35, *P* < .00001 [95% CI, 0.25-0.50]). Throughout these studies, variation was substantial (*I*^2^ = 71%).

### Saline vs Silicone Implants

**Figure 5.**
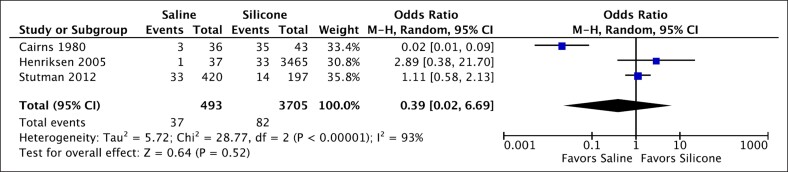
Forest plot for saline vs silicone.

Three studies (4198 cases) provided data on capsular contracture comparing saline vs silicone implants.^25,36,43^ There was no statistically significant difference between silicone and saline breast implants in capsular contracture rates (Figure 5, OR = 0.39, *P* = .52 [95% CI, 0.02-6.69]). Dispersion among these studies was considerable (*I*^2^ = 93%).

## DISCUSSION

Surgeons have many options to consider in the context of implant-based breast augmentation because surgical outcomes and rates of capsular contracture may vary depending on surgical technique and type of prosthesis used.^44,45^ This meta-analysis investigates the presence and strength of correlation between implant characteristics, including surface texture (smooth vs textured), plane of implant placement (prepectoral vs subpectoral placement of the implant), and implant filler material (saline vs silicone) on capsular contracture rates after implant-based breast augmentation. The Baker grade was used across all included studies to define capsular contracture, and only incidences of contracture with Baker Grades III and IV were considered as clinically significant.^4^

### Smooth vs Textured Implants

Smooth implants were associated with higher rates of capsular contracture when compared with textured implants (OR = 2.80, *P* < .00001 [95% CI, 1.92-4.08]), with substantial variation across these studies (*I*^2^ = 65%). This result is consistent with 3 previous analyses^8,46,47^ that found that textured implants reduce the incidence of postaugmentation capsular contracture when compared with smooth implants. It contrasts results from 1 systematic review^48^ where the authors found no statistically significant relationship between surface texture and incidence of capsular contracture when both implants were placed submuscularly.

The fact that lower rates of capsular contracture in the use of textured implants may be explained through the mechanism in which textured implants enhance adhesion on the implant surface was suggested in previous research. Valencia-Lazcano et al^49^ suggest that the greater surface area of textured implants results in the upregulation of adhesion-related genes in breast-derived fibroblasts, thereby reducing excessive motion of the implant that can provoke the overproduction of collagen from fibroblasts and ultimately leading to contracture. The researchers in other studies also suggested that textured implants can disrupt the contractile forces of myofibroblasts, leading to forces that are ineffective at inducing clinically relevant capsular contracture.^50-52^

### Subpectoral vs Prepectoral Placement of the Implant

This meta-analysis found that subpectoral placement of the implant was associated with significantly lower capsular contracture rates after breast augmentation compared with prepectoral placement (OR = 0.35, *P* < .00001 [95% CI, 0.25-0.50]), with substantial variation across these studies (*I*^2^ = 71%). These results are consistent with a previous meta-analysis.^53^ Shen et al^54^ observed that subglandular implant placement, a prepectoral technique, significantly increased the incidence of capsular contracture compared with subpectoral placement. However, their study did not find a significant difference when comparing subpectoral placement to subfascial placement, another form of prepectoral placement.

Numerous theories have been posited to explain why subpectoral placement of the implant may reduce the risk of capsular contracture compared with prepectoral placement. One prominent theory is that subpectoral placement better preserves the blood supply of the pectoralis major, thereby mitigating the risk of potential infection as well as providing cushioning and flexibility for the implant in the submuscular pocket.^55^ Additionally, the pectoralis major muscle provides greater coverage than the superficial fascia used in prepectoral placement, which may restrict movement of the implant and may improve adhesion, lessening the risk of contracture.^11^ Benito-Ruiz et al^37^ endorsed the same theory that the pocket created in prepectoral implant placement results in greater laxity and thus less adherence to the implant, possibly contributing to contracture. Further, as part of the “14-point plan” introduced by Adams et al, it has been shown that submuscular placement has anatomic advantages, which include avoidance of contact with breast parenchyma, as well as minimization of contact between the implant and bacteria native to breast ducts.^56,57^

### Saline vs Silicone Implants

This meta-analysis found no statistically significant difference between rates of capsular contracture after breast augmentation when comparing silicone and saline implants (OR = 0.39, *P* = .52 [95% CI, 0.02-6.69]), with considerable variation across these studies (*I*^2^ = 93%). These results contradict the findings of another meta-analysis,^45^ where the authors reported significantly higher rates of capsular contracture with silicone implants. However, 3 of the 4 comparative studies included in the prior meta-analysis were published before 1985, indicating that the silicone implants in question were of an older generation of implants. Further, 2 of the 4 studies of the prior meta-analysis reported firmness as an outcome, rather than using the traditional Baker classification. In a separate retrospective review,^55^ the researchers revealed that capsular contracture was nearly 8 times more likely following augmentation with saline implants compared with silicone implants. However, data regarding the volume of the saline implants were not reported, potentially confounding the relationship and leading to an inflated incidence of contracture.

The authors of previous research have indicated that in some instances, silicone implants will produce significant inflammatory responses because of silicone leakage through an intact membrane.^58^ Highly cohesive silicone implants have been shown to have lower leakage compared with low-cohesive ones and could potentially lead to lower capsular contracture rates.^57^ Many of the silicone implants examined in the study by El-Sheikh et al^45^ belonged to the second generation of silicone gel implants. The thin and seamless shells of these implants had similar rates of capsular contracture as first-generation implants and were also associated with a greater incidence of leakage of silicone particles into the body.^59^ These implants lacked the specifications and technological advancements now mandated by the FDA. To further support this theory, Prantl et al^60^ found that the severity of inflammatory response was associated with both greater capsular thickness and higher Baker grades after augmentation. Conversely, there is currently an absence of literature that addresses the potential mechanism in which saline implants may cause an increased incidence of capsular contracture, as seen in Blount et al.^55^

### Limitations

Limitations of this meta-analysis include the small number of studies comparing silicone vs saline breast implants. Additionally, most of the included studies were non-randomized trials, with a low level of evidence and a significant degree of heterogeneity. Some of the included studies had follow-up times of 12 months,^20,23,29,31^ or did not explicitly state the follow-up period.^21,24,28,32,39,43^ Capsular contracture is a progressive phenomenon, accumulating risk over time following surgery;^61^ hence, more long-term studies are required to make definite conclusions. Several other factors that were not considered could affect capsular contracture rates, such as surgical techniques (eg, transaxillary and periareolar), antibiotic irrigation of the breast pocket, and type of mesh or acellular dermal matrix use. The use of meshes for lower pole support is becoming more common.^62^ Additional studies will be needed to determine whether this affects capsular contracture rates. In this review, data from subfascial and subglandular planes were grouped together in the prepectoral group. However, a previous meta-analysis found that the subfascial plane is associated with lower rates of capsular contracture compared with the subglandular plane.^63^ Moreover, half of the studies included in this review were conducted in the 1980s and 1990s. Hence, this meta-analysis did not fully account for the significant change in breast implant handling and surgical technique over the last decade. Although most of the included studies reported data as the number of implants, 8 studies included data as the number of patients.^22,24,25,33,34,37-39^

## CONCLUSIONS

This meta-analysis suggests that textured-surface implants are associated with lower rates of capsular contracture after breast augmentation when compared with smooth-surface implants. Additionally, implants placed in the subpectoral plane may be associated with a statistically significant reduction in the rates of capsular contracture after breast augmentation, compared with implants placed in the prepectoral plane. There were no statistically significant differences in capsular contracture rates between saline and silicone-filled implants. However, it is important to recognize that the overall level of evidence of the included studies was low because of the lack of large randomized clinical trials, underscoring the need for caution in interpreting the findings of this meta-analysis. Augmentation mammaplasty remains one of the most common aesthetic procedures performed around the world; therefore, it is critical to continuously and tirelessly evaluate its outcomes through more standardized research to ensure not only optimization and longevity of the aesthetic results but also improvement in the patient safety and reduction in the number of reoperations.

## Supplemental Material

This article contains supplemental material located online at https://doi.org/10.1093/asjof/ojaf003.

## Supplementary Material

ojaf003_Supplementary_Data
