# Supplementary material for: Capsular Contracture After Breast Augmentation: A Systematic Review and Meta-Analysis
Source: Aesthet Surg J Open Forum. 2025 Jan 15;7:ojaf003. doi: 10.1093/asjof/ojaf003 (PMC11842228; doi:10.1093/asjof/ojaf003)
Supplement: ojaf003_Supplementary_Data [file ojaf003_supplementary_data.zip › Figure, Supplementary Digital Content 2.pdf]

	Random sequence generation (selection bias)	Allocation concealment (selection bias)	Blinding of participants and personnel (performance bias)	Blinding of outcome assessment (detection bias)	Incomplete outcome data (attrition bias)	Selective reporting (reporting bias)	Vested interest
Asplund 1996	+	+	+	+	+	+	?
Benito-Ruiz 2017	−	−	−	−	+	+	+
Burkhardt 1994	−	−	−	−	+	+	+
Burkhardt 1995	+	+	+	−	+	+	+
Cairns 1980	−	−	−	−	+	+	?
Calobrace 2018	−	−	−	−	+	+	+
Coleman 1991	+	+	+	+	+	+	+
Ersek 1991	−	−	−	−	+	+	+
Filiciani 2022	−	−	−	−	+	+	+
Hakelius 1992	−	−	−	−	+	+	?
Henriksen 2005	−	−	−	−	+	+	?
Khan 2013	−	−	−	−	+	+	?
Lista 2020	−	−	−	−	+	+	+
Malata 1997	+	+	+	+	+	+	+
Pereira 2009	−	−	−	−	+	+	?
Poeppl 2007	−	−	−	−	+	+	?
Pollock 1993	−	−	−	−	+	+	+
Puckett 1987	−	−	−	−	+	+	?
Shi 2015	−	−	−	−	+	+	+
Spear 2014	−	−	−	−	+	+	−
Stevens 2013	−	−	−	−	+	+	?
Stutman 2012	−	−	−	−	+	+	−
Tarpila 1997	+	+	+	+	+	+	?
Vazquez 1987	−	−	−	−	+	+	?
