# Supplementary material for: Capsular Contracture After Breast Augmentation: A Systematic Review and Meta-Analysis
Source: Aesthet Surg J Open Forum. 2025 Jan 15;7:ojaf003. doi: 10.1093/asjof/ojaf003 (PMC11842228; doi:10.1093/asjof/ojaf003)
Supplement: ojaf003_Supplementary_Data [file ojaf003_supplementary_data.zip › Table, Supplementary Digital Content 1.docx]

**Table, Supplemental Digital Content 1. Detailed search strategy.**

| **Database** | **Time span** | **Search strategy** |
| --- | --- | --- |
| PubMed MEDLINE | January 1947 to 25 December 2023 | ((breast reconstruction[MeSH Terms]) OR (breast implant[MeSH Terms])) OR ((breast*) AND ((implant*) OR (reconstruct*)))) AND ((((subpectoral OR submuscular OR retropectoral) AND (prepectoral OR suprapectoral OR subcutaneous OR premuscular OR supramuscular OR muscle-sparing OR pectoralis-sparing)) OR ((textur*) AND (smooth))) OR ((saline) AND (silicone))) |
| EMBASE (OvidSP) | January 1974 to 25 December 2023 | 1 breast reconstruction/  2 breast  3 implant* OR reconstruct*  4 3 AND 4  5 1 OR 4  6 subpectoral OR submuscular OR retropectoral  7 prepectoral OR suprapectoral OR subcutaneous OR premuscular OR supramuscular OR muscle-sparing OR pectoralis-sparing  8 6 AND 7  9 textur* AND smooth  10 saline AND silicone  11 8 OR 9 OR 10  12 5 AND 11 |
| Cochrane library | January 1974 to December 2023 | 1 breast reconstruction/  2 breast  3 implant* OR reconstruct*  4 3 AND 4  5 1 OR 4  6 subpectoral OR submuscular OR retropectoral  7 prepectoral OR suprapectoral OR subcutaneous OR premuscular OR supramuscular OR muscle-sparing OR pectoralis-sparing  8 6 AND 7  9 textur* AND smooth  10 saline AND silicone  11 8 OR 9 OR 10  12 5 AND 11 |
